# Development of a multiplex PCR to detect and discriminate porcine circoviruses in clinical specimens

**DOI:** 10.1186/s12879-019-4398-0

**Published:** 2019-09-05

**Authors:** Keli Yang, Zuwu Jiao, Danna Zhou, Rui Guo, Zhengying Duan, Yongxiang Tian

**Affiliations:** 1 Institute of Animal Husbandry and Veterinary, Hubei Academy of Agricultural Sciences, Wuhan, 430064 People’s Republic of China; 2 Key Laboratory of Prevention and Control Agents for Animal Bacteriosis (Ministry of Agriculture), Wuhan, 430064 People’s Republic of China; 3 Hubei Key Laboratory of Animal Embryo and Molecular Breeding, Wuhan, 430064 People’s Republic of China

**Keywords:** Porcine circovirus, PCV1, PCV2, PCV3, Viral discrimination, Multiplex PCR

## Abstract

**Background:**

A diagnostic method to simultaneously detect and discriminate porcine circovirus type 1 (PCV1), porcine circovirus type 2 (PCV2) and porcine circovirus type 3 (PCV3) in clinical specimens is imperative for the differential diagnosis and monitoring and control of PCVs in the field.

**Methods:**

Three primer pairs were designed and used to develop a multiplex PCR assay. And 286 samples from 8 farms in Hubei province were tested by the developed multiplex PCR assay to demonstrate the accuracy.

**Results:**

Each of target genes of PCV1, PCV2 and PCV3 was amplified using the designed primers, while no other porcine viruses genes were detected. The limit of detection of the assay was 10 copies/μL of PCV1, PCV2 OR PCV3. The results of the tissue samples detection showed that PCV1, PCV2 and PCV3 are co-circulating in central China. The PCV1, PCV2 and PCV3 singular infection rate was 52.4% (150/286), 61.2% (175/286) and 45.1% (129/286), respectively, while the PCV1 and PCV2 co-infection rate was 11.2% (32/286), the PCV1 and PCV3 co-infection rate was 5.9% (17/286), the PCV2 and PCV3 co-infection rate was 23.4% (67/286), and the PCV1, PCV2 and PCV3 co-infection rate was 1.7% (5/286), respectively, which were 100% consistent with the sequencing method and real-time PCR methods.

**Conclusions:**

The multiplex PCR assay could be used as a differential diagnostic tool for monitoring and control of PCVs in the field. The results also indicate that the PCVs infection and their co-infection are severe in Hubei province, Central China.

## Background

**Fig. 1:**
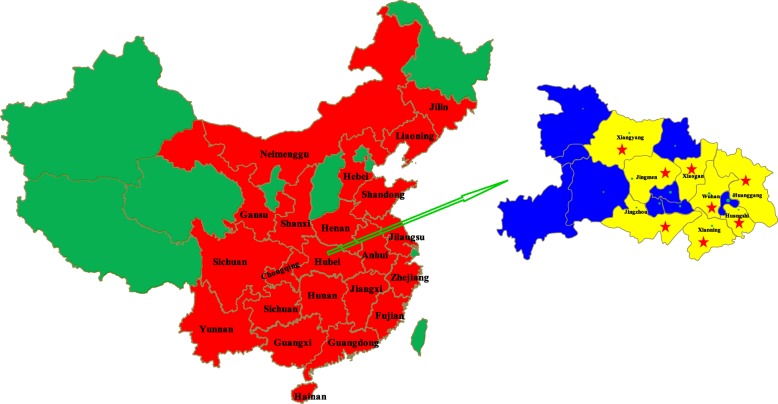
Geographical distribution of PCV3 in China (red regions, till June 2018) and the position of pig farms (red stars) in this study

Porcine circoviruses (PCVs), are non-enveloped and circular DNA viruses, which belong to the genus *Circovirus*, family *Circoviridae* [1]. At present, PCVs are smallest animal viruses. Two strains of circovirus, PCV1, PCV2, had been proved as infectious to pigs before 2015 [2]. PCV1 was first isolated and in 1974, which just was a contaminant of the PK-15 cell, and nonpathogenic for pigs [3, 4]. However, PCV1 was found that it can replicate efficiently and produce pathology in the lungs of porcine fetuses and have a certain impact on porcine alveolar macrophages [5]. It is a potential damage to the immune system of piglets. PCV2 was first identified from the pigs which was suffering post-weaning multisystemic wasting syndrome (PMWS) in the middle of 1990s [6]. Pigs infected PCV2 have various clinical diseases, which have made the swine industries huge economic losses all over the world [7]. In 2016, a novel circovirus, called PCV type 3 (PCV3), was isolated from diseased pigs in the USA [8, 9]. Subsequently, several outbreaks of it were reported from the United Kingdom [10], Poland [11], Italy, Denmark, Spain [12], Korean [13], Brazil [14], and China [2, 15–17]. PCV1 [5] and PCV3 [2, 8, 12, 13] had been confirmed as potential pathogen associated with many kinds of clinical symptoms, which are similar as PCV2 infection*.* And now, PCV3 has been found in about 20 provinces in China (Fig. 1).

Both PCV1 and PCV2 infections are common in pig herds all over the world [18], and PCV3 is the third porcine circovirus type found in swine, which is circulating in the swine population [8]. The co-infection of PCV3 with PCV2 was reported in clinical samples of diseased pigs in Hubei province [2]. And co-infection of PCV2 with PCV1 was found in Hubei province [19]. However, PCV1, PCV2 and PCV3 or their co-infections were tested separately using different methods in the previous reports. Considering the high impact of PCV2 and PCV3 on the economy of pig industry, the impact of the potential pathogenic-PCV1, and the similarities between the clinical manifestations associated with PCV3 and PCV2, it is necessary to develop a convenient, sensitive, and specific diagnostic approach to discriminate PCV1, PCV2 and PCV3 infection.

However, there is no convenient and specific diagnostic assay capable of differentiating PCV1, PCV2 and PCV3 infection. Therefore, in present study, a, simple, specific and sensitive multiplex PCR assay was developed to detect and discriminate PCV1, PCV2 and PCV3 in clinical specimens. The accuracy and applicability of the multiplex PCR were evaluated for detection of PCVs DNA in clinical samples collected from the eight pig farms in Hubei province (Fig. 1) where co-infection of PCVs was reported [2].

## Methods

### Cells and viruses

PK15 cells were cultured in DMEM supplemented with 10% fetal bovine serum (Gibco) at 37 °C in an incubator with humidified 5% CO_2_. PCV1, PCV2, PCV3 and other viruses, including Torque teno sus viruse type 1 (TTSuV1), Torque teno sus viruse type 2 (TTSuV2), pseudorabies virus (PRV), porcine parvovirus (PPV), rotavirus (RV), Japanese encephalitis virus (JEV), porcine epidemic diarrhea virus (PEDV), porcine deltacorona virus (PDCoV), stored at Key Laboratory of Prevention and Control Agents for Animal Bacteriosis (Ministry of Agriculture), were used to verify the specificity of the developed multiplex PCR assay. And the viruses PCV1, PCV2, PCV3 were also used as positive control viruses. Viral genomic DNA/RNA for the specificity of the proposed multiplex PCR assay were extracted according to our previous study [20].

### Primers design

The multiple sequences in GenBank for each virus were aligned including PCV-1 (*n* = 12), PCV-2 (*n* = 12), and PCV-3 (*n* = 12) that covers 85.71, 1.07, and 5.36% of the sequences in GenBank for the respective virus. The nucleotide sequences were submitted to BLAST using MEGA 5.10 software. Three pairs of primers were designed based on the characteristic of the PCV strains (Table 1). The PCR-amplified PCV1, PCV2 and PCV3 products were 310, 505 and 1021 bp, respectively. The primers were synthesized by Sangon Biotech Co., Ltd. (Shanghai, China).

**Table 1:** Sequences of the primers for multiplex PCR

Primer	Primer sequences (5’-3’)	Origin/target gene	Location	Products
PCV1-F	GAAAGTGAGCGGGAAGAT	PCV1 (GenBank: KX827790.1) /Rep	499–516	310 bp
PCV1-R	CTGATTGCTGGTAATCAA		790–808	
PCV2-F	CACATCGAGAAAGCGAAAGGAAC	PCV2(GenBank: MG229682.1) /Rep	294–316	505 bp
PCV2-R	TGCGGGCCAAAAAAGGTACAGTT		776–798	
PCV3-F	AGCAGTGCTCCCCATTGA	PCV3(GenBank: KX898030.1) /Rep, Cap	1431–1448	1021 bp
PCV3-R	TGGGCCCGACCAAATCCGG		428–446	

### DNA/RNA extraction

Viral DNA/RNA samples for proposed multiplex PCR were extracted using the Viral DNA/RNA Miniprep Kit (Axygen Scientific, CA, USA) from the tissue samples. Total DNA/RNA was eluted with 30 μL of diethyl pyrocarbonate-treated water, used immediately or stored at −80 °C. cDNA was constructed by M-MLV reverse transcriptase (Promega, Madison, WI, USA) with 5–7 μL RNA (RV, JEV, PEDV and PDCoV) .

### Single PCR and the standard templates construction

The PCR was performed in a 50 μL reaction mixture including 5.0 μL 10 × PCR buffer, 4 μL dNTPs (10 mM of each), 1.0 μL primer (10 μM), 2.5 U Taq DNA polymerase (5 U/μL) (TaKaRa, Dalian, China), 3 μL DNA (100 copies/μL), and added distilled water to 50 μL.

The amplifications were performed under the following conditions: after initial denaturation at 95 °C for 4 min, 35 cycles were conducted at 94 °C for 30 s, 55 °C for 40 s and 72 °C for 60 s, followed by a final extension at 72 °C for 10 min. Each specific viral target fragment was cloned into the plasmid pMD18-T (TaKaRa). The constructed recombinant plasmids were sequenced and confirmed to use as standard templates for optimization of the following PCR assays.

### Optimization of the multiplex PCR

In order to obtain the best reaction parameters, the multiplex PCR was optimized by varying single parameters while other parameters were maintained.The optimization was performed in a 50 μL PCR reaction mixture as follows: 10 × PCR buffer 5.0 μL, 10 mM dNTPs 2–4 μL, each 10 μM primer (Table 1) 0.5–1 μL, Taq DNA polymerase (5 U/μL) (TaKaRa, Dalian, China) 0.5–1 μL, the DNA template 3.0–5.0 μL(100 copies/μL), and added distilled water to 50 μL.

The amplifications were performed under the following conditions in a thermal cycler (Bio-Rad, Hercules, CA, USA). After 5 min initial denaturation at95 °C, 35 cycles were conducted at 94 °C for 40 s, 52–58 °C for 40 s and 72 °C for 50–70 s, followed by a 10-min final extension at 72 °C. The PCR products were detected according to our previous study [20].The specific viral target fragments were clonedinto the plasmid pMD18-T (TaKaRa, Dalian, China).

Plasmids containing the PCV1, PCV2 or PCV3 gene were purified using a MiniBEST Plasmid Purification Kit (TaKaRa, Dalian, China). Each plasmid sample concentration was determined by measuring the absorbance at 260 nm using a Eppendorf BioSpectrometer (Eppendorf, Hamburg, Germany), and each cloned gene copy number was quantified as previously described [21]. The standard PCV1, PCV2 and PCV3 DNA samples were ten-fold diluted (from 10^7^ to 10^−2^copies/μL) and stored at − 80 °C until use.

### Specificity of the multiplex PCR

Specificity of the multiplex PCR assay was determined by using the DNA or cDNA of above-mentioned porcine viruses as templates and ddH_2_O as a negative control. All templates and ddH_2_O were repeated three times for specificity of the multiplex PCR. PCV1, PCV2 and PCV3 were verified by sequencing and the other virus strains (TTSuV1, TTSuV2, PRV, PPV, RV, JEV, PEDV and PDCoV) were identified by serological or PCR methods.

### Sensitivity of the multiplex PCR

Total DNA from the plasmids was extracted and used to detect the sensitivity of the developed multiplex PCR assay. The ten-fold diluted standard PCV1, PCV2 or PCV3 DNA samples (from 10^7^ to 10^−2^copies/μL) were used as templates for the multiplex PCR. All samples were repeated in triplicate for sensitivity of the multiplex PCR.

### Interference test of the multiplex PCR

To investigate whether the different viral concentrations could influence the detection efficiency of this multiplex PCR assay, 10-fold serial dilutions (10–1–102 copies/μL) in distilled water were prepared to extract the PCVs DNA. Two combinations were tested: (i) the PCR mixture containing three primer pairs and three templates containing equal volumes of DNA from different dilutions, and (ii) three primer pairs and three templates containing equal volumes of DNA from same dilutions.

### Clinical sample collection

A total of 286 tissue samples including lung, spleen and lymph node, were collected from diseased pigs from eight farms in Hubei Province (Fig. 1), central China, from October, 2017 to March, 2018. They are commercial source. They were collected and sent to us by the owners to detect PCVs. These pigs were suspected to have clinical signs of PCVs. Specifically, the clinical signs of the diseased pig were as follows: 92 samples of reproductive failure cases, 78 samples of pigs with PMWS, 65 samples of respiratory disorders cases, 36 diarrhea samples and 15 PDNS samples. All samples were collected in accordance with the standards for animal welfare approved by the Animal Ethics Committee of the Hubei province.

### Sample preparation

The tissue samples were cut into pieces in a homogenizer, added five times quality of phosphate-buffered saline (PBS, 0.1 M, pH 7.2) and homogenized. All samples were frozen and thawed three times, and centrifuged at 12,000 rpm for 5 min at 4 °C. The supernatants were used for DNA extraction immediately or stored at − 80 °C until use.

### Detection of PCVs

The DNA of the clinical specimens were detected using the multiplex PCR assay to investigate the epidemiology of PCVs in Hubei province, central China. To confirm the accuracy of the developed protocol, each PCR product of positive samples was cloned into the plasmid pMD18-T and sequenced. And two real-time PCR methods [22, 23] were used to detect PCV1, PCV2 and PCV3 for comparison.

### Animal experiment

Twenty PCV free pigs were average divided into four groups randomly, PCV1, PCV2 and PCV3 experiment group and control group. The pigs are experimental source. They were derived from the farm of Institute of Animal Husbandry and Veterinary, Hubei Academy of Agricultural Sciences. Each pig of the experiment group was injected with 1 mL PCV1, PCV2 or PCV3 inoculums (>10^6.0^TCID_50_/mL) intravenously, respectively. The control group was injected with 1 mL of sterile physiological saline. Each group was fed separately. Two weeks later, all the pigs were euthanized (CO_2_ inhalation) to collected tissue samples. Viral DNA was extracted as described above and used as templates to detect PCV1, PCV2 or PCV3 using the established multiplex PCR.

## Results

### Optimization of the multiplex PCR assay

The optimum parameters of the proposed multiplex PCR were as follows. A final 50-μL volume of master mix for the multiplex PCR including 10 × Buffer 5.0 μL, Taq polymerase (5 U/μL) 0.5 μL, DNA template 5.0 μL, dNTPs (10 mM) 4 μL, each primer (10 μM) 1.0 μL, and nuclease-free water was added to make a total volume of 50 uL per reaction. An optimized experimental protocol consisted of a 5-min denaturation program at 95 °C, and 35 cycles amplification program (denaturation at 94 °C for 40 s, 56 °C for 40 s, and elongation at 72 °C for 50 s), followed by an extension at 72 °C for 10 min.

**Fig. 2:**
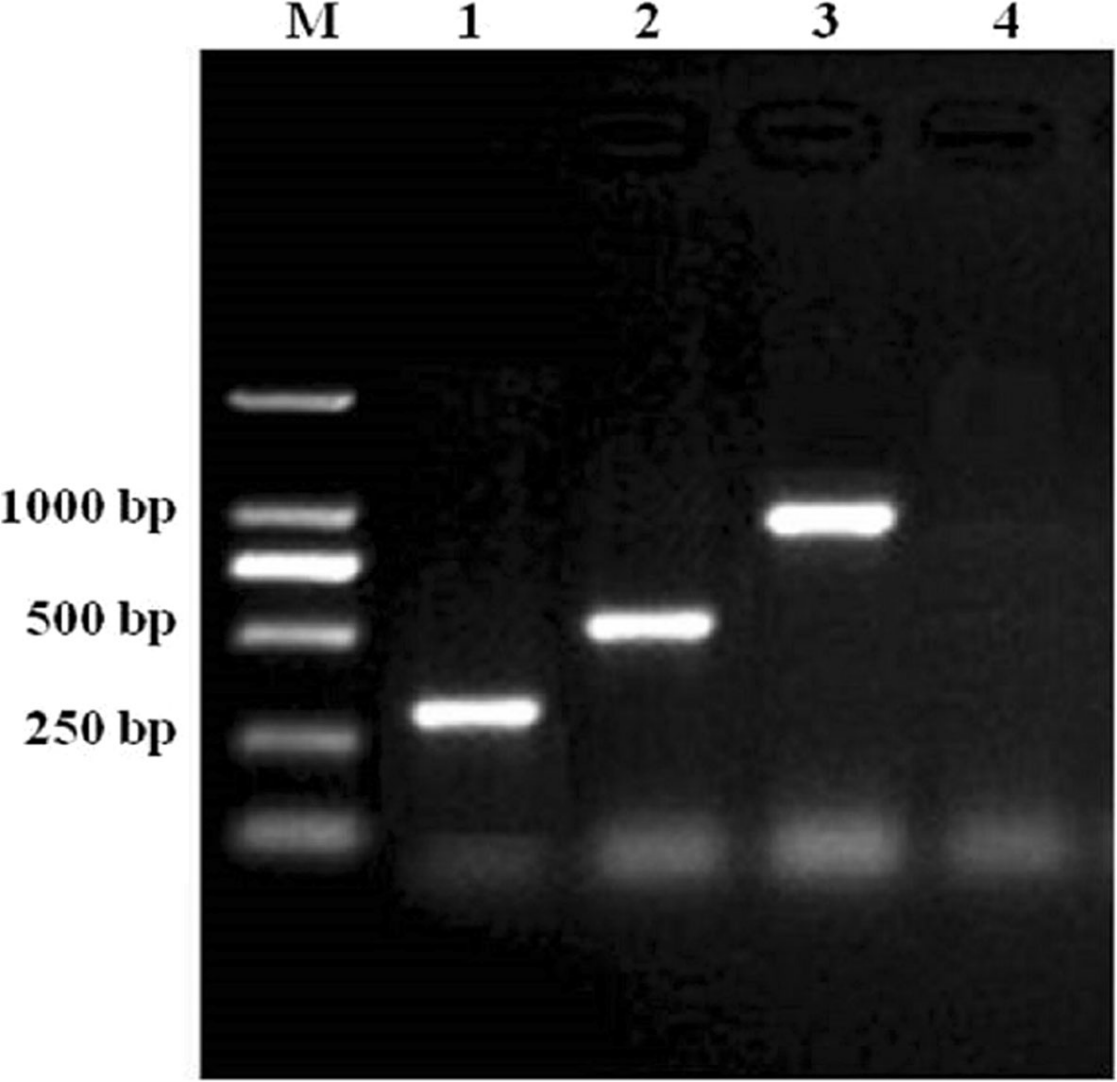
Electrophoresis of multiplex PCR products in optimization conditions. Lane M, DL2000 DNA marker; lane 1, PCV1; lane 2, PCV2; lane 3, PCV3; lane 4, ddH_2_O

DNAs of PCV1, PCV2, PCV3, and ddH_2_O, the negative control, were detected using the protocol described above and the multiplex PCR amplification results are illustrated in Fig. 2.

### Specificity of the proposed multiplex PCR

**Fig. 3:**
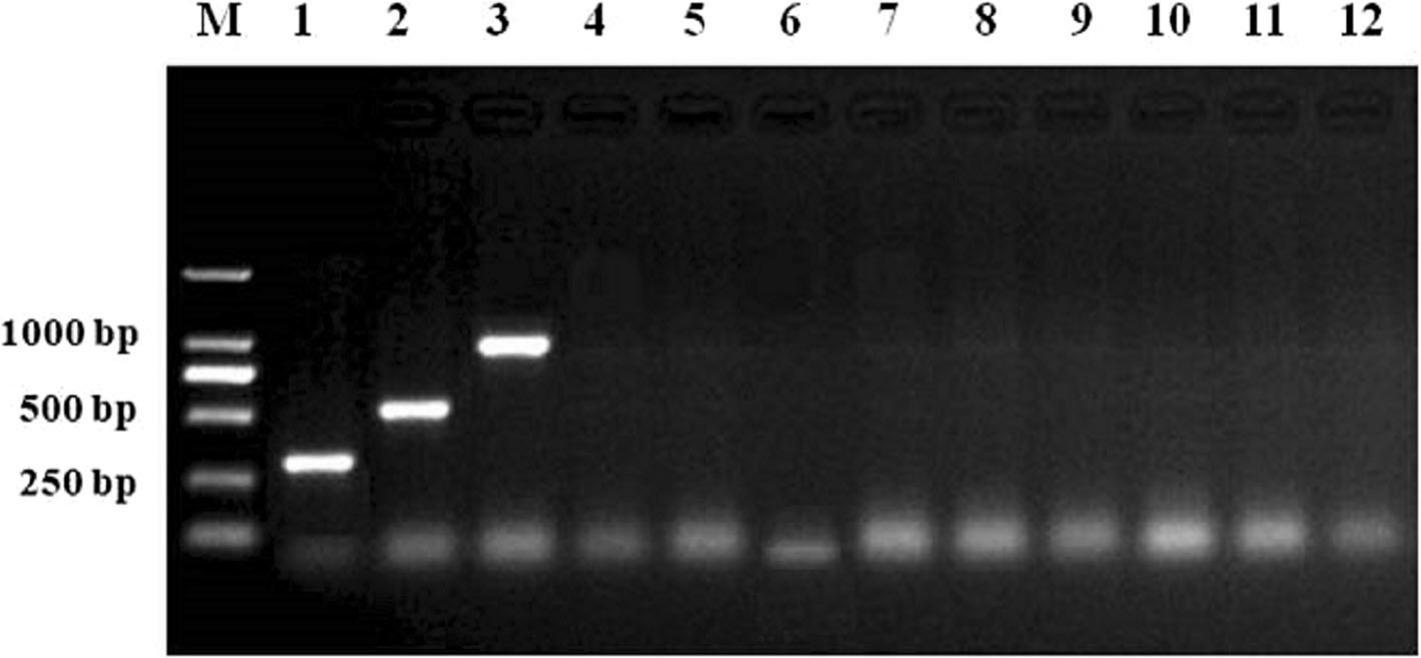
Specificity of multiplex PCR for the detection of PCVs. Lane M, DL2000 DNA marker; lane 1, PCV1; lane 2, PCV2; lane 3, PCV3; lane 4, TTSuV1; lane 5, TTSuV2; lane 6, PRV; lane 7, PPV; lane 8, RV; lane 9, JEV; lane 10,PEDV; lane 11, PDCoV; lane 12, ddH_2_O

The specificity of the three primer pairs for PCVs was analyzed using the developed multiplex PCR. Each DNA/cDNA of the virus mentioned above was amplified using the three defined primer pairs in a reaction respectively. As illustrated in Fig. 3, the multiplex PCR assay was specific for PCVs because no amplification products occurred with TTSuV1, TTSuV2, PRV, PPV, RV, JEV, PEDV, PDCoV and ddH_2_O (lanes 4–12), whereas the PCV1, PCV2 and PCV3 target genes were specifically amplified using the three defined primer pairs (lanes 1–3).

### Sensitivity of the proposed multiplex PCR

**Fig. 4:**
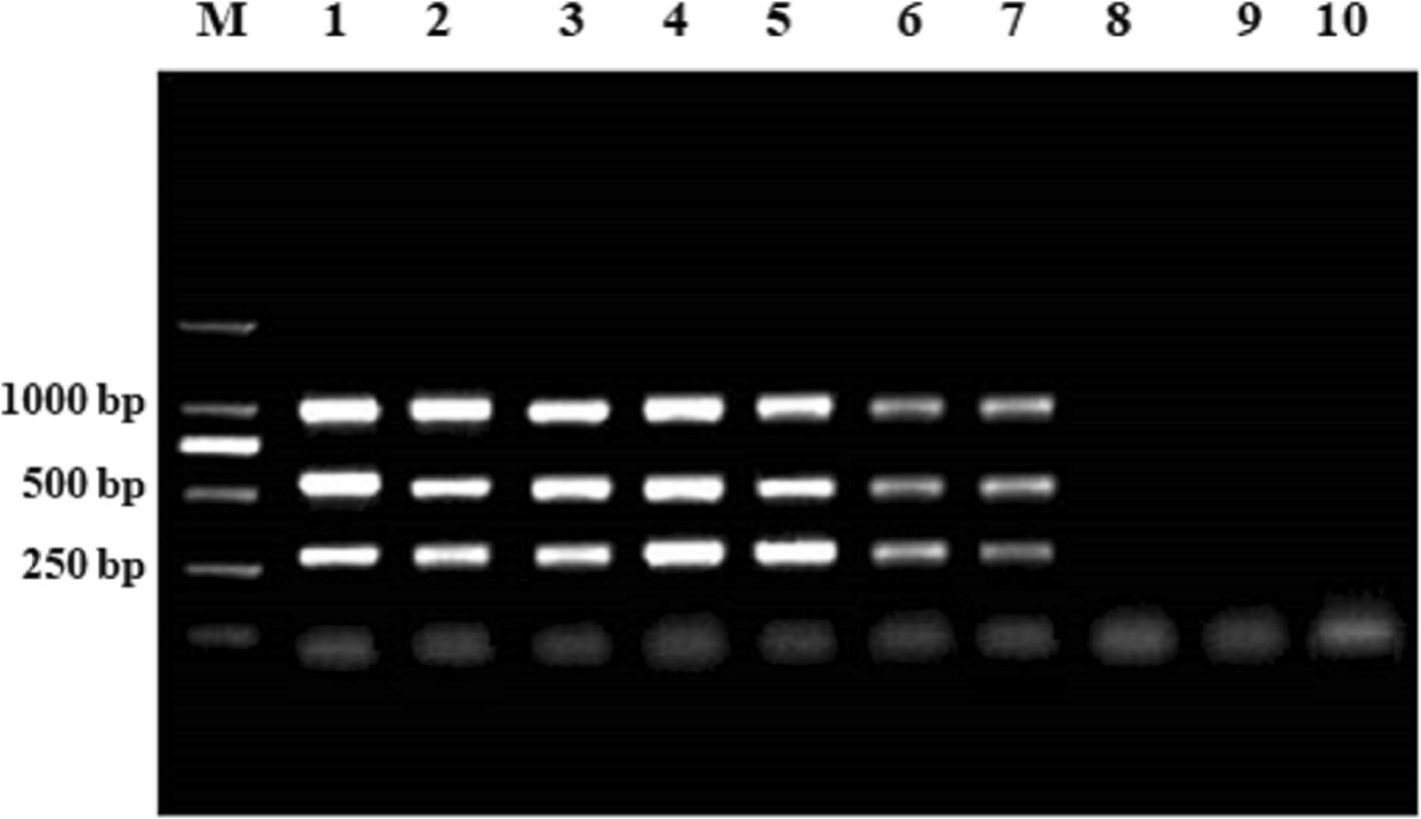
Sensitivity of multiplex PCR for the detection of PCVs. Lane M, DL2000 DNA marker; lanes 1–10 are: 1, 10^7^; 2, 10^6^; 3, 10^5^; 4, 10^4^; 5, 10^3^; 6, 10^2^; 7, 10^1^; 8, 10^0^; 9, 10^− 1^; 10, 10^− 2^ copies/μL

The sensitivity of the proposed multiplex PCR assay was defined as the minimum DNA molecule concentration which could be detected. DNA standards, which were10-fold diluted, with known copy numbers (10^7^ copies/μL to 10^− 2^ copies/μL) were used for the multiplex PCR. As shown in Fig. 4, the detection limit of the multiplex PCR was 10 copies/μL plasmid DNA molecules for PCV1, PCV2 or PCV3, which indicated that the sensitivity of the multiplex PCR was 10 copies/μL for PCV1, PCV2 and PCV3.

### Interference test of the multiplex PCR

A mixture of different concentrations of the three PCV strains was amplified using the multiplex PCR. The results showed that detection of the three PCV strains was not influenced if the concentration of them was similar (Table 2).

**Table 2:** Results of interference test of the multiplex PCR

Virus concentration (copies/μL)			Multiplex PCR results			
PCV1	PCV2	PCV3	PCV1	PCV2	PCV3	
10^2^	10^1^	10^0^	+	+	–	
10^1^	10^0^	10^−1^	+	–	–	
10^0^	10^−1^	10^−2^	–	–	–	
10^− 1^	10^− 2^	10^2^	–	–	+	
10^2^	10^2^	10^2^	+	+	+	
10^1^	10^1^	10^1^	+	+	+	
10^0^	10^0^	10^0^	–	–	–	
10^−1^	10^−1^	10^−1^	–	–	–	

+, positive result for multiplex PCR test; −, negative result for multiplex PCR test

### Detection of viruses in clinical specimens

A total 286 clinical samples were tested by the multiplex PCR assay. The results were as follows: The PCV1-positive, PCV2-positive and PCV3-positive rate at the farm level was 62.5% (5/8), 87.5% (7/8) and 62.5% (5/8), respectively. The positive rates of PCV1, PCV2 and PCV3 in these samples were 52.4% (150/286), 61.2% (175/286) and 45.1% (129/286), respectively, which were 100% consistent with the sequencing method and the real-time PCR methods (Table 3). The results of the multiplex PCR method and subsequent sequencing further demonstrated the accuracy of the developed assay.

**Table 3:** Detection of clinical specimens by multiplex PCR, sequencing method and real-time PCR

Pig Farm	No. of specimens	multiplex PCR			sequencing method			real-time PCR			Concordance rate (%)
		PCV1 positive (%)	PCV2 positive (%)	PCV3 positive (%)	PCV1 positive (%)	PCV2 positive (%)	PCV3 positive (%)	PCV1 positive (%)	PCV2 positive (%)	PCV3 positive (%)	
1	50	0 (0)	36 (72.0)	33 (66.0)	0 (0)	36 (72.0)	33 (66.0)	0 (0)	36 (72.0)	33 (66.0)	100
2	48	41 (85.4)	33 (68.8)	31 (64.6)	41 (85.4)	33 (68.8)	31 (64.6)	41 (85.4)	33 (68.8)	31 (64.6)	100
3	38	35 (92.1)	25 (65.8)	0 (0)	35 (92.1)	25 (65.8)	0 (0)	35 (92.1)	25 (65.8)	0 (0)	100
4	36	32 (88.89)	27 (75.0)	26 (72.2)	32 (88.89)	27 (75.0)	26 (72.2)	32 (88.89)	27 (75.0)	26 (72.2)	100
5	33	0 (0)	21 (63.6)	20 (60.6)	0 (0)	21 (63.6)	20 (60.6)	0 (0)	21 (63.6)	20 (60.6)	100
6	30	0 (0)	17 (56.7)	19 (63.3)	0 (0)	17 (56.7)	19 (63.3)	0 (0)	17 (56.7)	19 (63.3)	100
7	26	20 (76.9)	0 (0)	0 (0)	20 (76.9)	0 (0)	0 (0)	20 (76.9)	0 (0)	0 (0)	100
8	25	22 (88.0)	16 (64.0)	0 (0)	22 (88.0)	16 (64.0)	0 (0)	22 (88.0)	16 (64.0)	0 (0)	100
Total	286	150 (52.4)	175 (61.2)	129 (45.1)	150 (52.4)	175 (61.2)	129 (45.1)	150 (52.4)	175 (61.2)	129 (45.1)	100

Additionally, the PCV1 and PCV2 co-infection rate was 11.2% (32/286), the PCV1 and PCV3 co-infection rate was 5.9% (17/286), the PCV2 and PCV3 co-infection rate was 23.4% (67/286), and the PCV1, PCV2 and PCV3 co-infection rate was 1.7% (5/286), respectively in the samples from eight pig farms (Table 4). The total co-infection rate was 42.3% (121/286) in all of the detected samples.

**Table 4:** Detection of the co-infection of clinical specimens by multiplex PCR

Pig Farm	No. of specimens	PCV1 and PCV2 positive (%)	PCV1 and PCV3 positive (%)	PCV2 and PCV3 positive (%)	PCV1, PCV2 and PCV3positive (%)	
1	50	0 (0)	0 (0)	20 (40.0)	0 (0)	
2	48	13 (27.1)	9 (18.8)	19 (39.6)	2 (4.2)	
3	38	9 (23.7)	0 (0)	0 (0)	0 (0)	
4	36	8 (22.2)	5 (13.9)	13 (36.1)	3 (8.3)	
5	33	0 (0)	0 (0)	5 (15.2)	0 (0)	
6	30	0 (0)	0 (0)	10 (33.3)	0 (0)	
7	26	0 (0)	0 (0)	0 (0)	0 (0)	
8	25	2 (8.0)	3 (12.0)	0 (0)	0 (0)	
Total	286	32 (11.2)	17 (5.9)	67 (23.4)	5 (1.7)	

### Detection of viruses in animals infected with PCV

The samples from the 20 pigs were tested by the multiplex PCR assay. The results were as follows: The PCV1-positive rate was 100% (5/5) in PCV1 group, the PCV2-positive rate was 100% (5/5) in PCV2 group, and the PCV3-positive rate was 100% (5/5) in PCV3 group, respectively. None has clinical signs in PCV1 group, while two fifths in PCV2 group and there fifths in PCV3 group present with clinical symptoms as dermatitis, respiratory symptoms, severe weight loss etc. (Table 5). The results showed that the developed assay can test the viruses in the samples from “non-clinical” pigs, and indicated that it is adaptable for testing field samples.

**Table 5:** Detection of the specimens from the infection pigs by multiplex PCR

Group	No. of specimens	PCV1 positive (%)	PCV2 positive (%)	PCV3 positive (%)	Clinical Signs	
PCV1	5	100	0	0	none	
PCV2	5	0	100	0	dermatitis, respiratory symptoms, severe weight loss (2/5)	
PCV3	5	0	0	100	dermatitis, respiratory symptoms, severe weight loss (3/5)	
Control	5	0	0	0	none	

## Discussion

PCV3 is associated with nephropathy syndrome, reproductive failure and porcine dermatitis [8], respiratory disease complex, and cardiac and multisystemic inflammation [9]. PCV2 is clinically characterized by decreased weight gain, wasting, dyspnea, and enlarged lymph nodes. It has also been identified from diseased pigs with various other clinical presentations, such as porcine dermatitis and nephropathy syndrome, porcine respiratory disease complex (PRDC) and reproductive failure [24]. PCV2 is confirmed as an endemic viral disease of swine all over the world. And in modern swine production, it is also recognized as one of the most economically important infectious pathogens [25]. Although PCV1-seroprevalence at herd level diversifies between 10 and 100% [26], it is generally believed that PCV1 has no pathogenicity to pigs [4, 6]. It was found originally as a contaminant of the porcine kidney cell line PK15 [3]. However, PCV1 has recently gained focus of attention because it was discovered as contaminant in several live veterinary vaccines and human vaccines [18, 27]. And PCV1 has been identified in cases of congenital tremors in aborted/stillborn piglets and newborn pigs. The results showed that PCV1 can proliferate and may produce pathological change in the lungs of fetal porcine [5]. And a new type of porcine circovirus in swine, a type 1 and type 2 PCV recombinant was isolated from swine samples [28]. There could be a potential damage to the piglets’ immune system caused by PCV1 infection [29]. Both PCV1 and PCV2 infections are common in pig herds worldwide [30], and PCV3 is already widely outbroken and distributed on pig farms in many countries [2, 8, 12, 13]. Single infection of PCV2 or PCV3, or co-infection of PCV1, PCV2 and PCV3 could cause many kinds of diseases in pig herds [2, 10, 13]. Veterinary workers should develop suitable prevention and control policies for PCVs and their novel strains emerging from viral evolution [31].

In order to establish prevention and control strategies for PCVs, a convenient and sensitive diagnostic method is necessary to simultaneously detect and discriminate PCVs in clinical samples. PCR assays are sensitive methods to detect a circovirus infection in viremia animals [32]. And The multiplex PCR assay is specific, rapid and easy to interpret the prevalence, epidemiology and infectious potential of some pathogens [33]. Several different PCR methods, including digital droplet PCR (ddPCR), real-time PCR and quantitative PCR (qPCR) using specific primers have been developed for detection of PCV1 and PCV2 [34–43]. And recently, two qPCR methods were proposed to detect and quantify PCV3 DNA [8, 44]. However, there is no specific, sensitive and reliable single assay capable of detecting and differentiating infection by PCV1, PCV2 and PCV3. Therefore, a specificand sensitive multiplex PCR assay to detect and discriminate PCV1, PCV2 and PCV3 in clinical specimens was developed in the present study.

In this study, an efficient and sensitive multiplex PCR assay was developed to detect and discriminate PCV1, PCV2 and PCV3 using three specific primer pairs. The sizes of the amplified PCR products of PCV1, PCV2 and PCV3 strain from the specific primers are very different, which can be easily differentiated by electrophoresis. The specific primers for the multiplex PCR assay successfully amplified the 310-bp PCV1, 505-bp PCV2 or 1021-bp PCV3 gene. Furthermore, the multiplex PCR assay with three sets of PCV1-, PCV2- and PCV3-specific primers simultaneously detected and discriminated PCV1, PCV2 and PCV3 DNA in a single reaction. And the sensitivity of the multiplex PCR assay was 10 copies/μL for PCV1, PCV2 and PCV3, which is similar to several previous studies [2, 34, 44, 45].

Although multiplex qPCR assays that can discriminate PCV2 and PCV3 have already been developed, including one study from China [46], but all the assays must be run in an expensive instrument—Fluorescent Quantatitive PCR. It is unaffordable for many laboratories especially those in the counties and towns in China, so the multiplex qPCR assays couldn’t be run in their laboratories. However, almost all of them are equipped with the conventional PCR instrumentation. The sensitivity of the multiplex PCR developed in this study is similar to several qPCR assays. Definitely, the multiplex PCR is a convenient and sensitive diagnostic method to detect and discriminate PCVs in clinical samples in China and any other developing countries.

A total of 286 specimens from eight pig farms in Hubei province, central China, were analyzed using the proposed multiplex PCR. The results were 100% same as those of sequencing method and real-time PCR methods. The PCV1, PCV2 and PCV3 singular infection rate, the PCV1 and PCV2 or PCV3 co-infection rate, the PCV2 and PCV3 or PCV1, PCV2 and PCV3 co-infection rate were higher than the previous reports [2, 16, 45]. The results indicated that the PCVs infection and their co-infection are severe in Hubei Province, Central China. And the epidemiology and genome characterizations of PCVs in Hubei Province would be further studied in the near future.

DNAs of PCV2 and PCV3 were detected using multiplex PCR in aborted fetal tissue samples and respiratory diseased piglet tissue samples. The results suggested that both PCV2 and PCV3 infection are associated with reproductive failure and respiratory disease at the infection pig farms, as previous researches [2, 8, 44]. The singular infection of PCV2 and PCV3, and PCV2, PCV3 and/or PCV1 co-infection play an etiological role in porcine circovirus associated disease (PCVAD), which had caused huge economic losses to pig farms all over the world.

The multiplex PCR assay is sensitive, and it can also simultaneously discriminate PCV1, PCV2, and PCV3 in a single reaction, which makes it lower cost and less time. It will be a useful tool to detect and discriminate PCVs in field samples. Considering the prevalence of PCV1, PCV2 and PCV3 co-infection in the field, the multiplex PCR will enable the correct diagnosis of suspected clinical cases and stimulate further epidemiological researches for its control. However, there would be any other PCV genotypes in pig farms. Hence, an accurater PCV identification which covers all types of PCV genotypes should be established in the near future.

## Conclusions

In conclusion, the developed multiplex PCR is a convenient, sensitive, efficient, and highly specific assay to detect and discriminate PCVs, which will be useful in etiological and epidemiological studies, as well as diagnosis in clinical cases. The accuracy and simplicity of the assay makes it a useful, suitable and powerful tool for PCVs detection, prevention and control in China and any other developing countries.

## Data Availability

The datasets used and/or analysed during the current study are available from the corresponding author on reasonable request.

## Abbreviations

JEV: Japanese encephalitis virus
PCV: Porcine circovirus
PCV1: Porcine circovirus type 1
PCV2: Porcine circovirus type 2
PCV3: Porcine circovirus type 3
PDCoV: Porcine deltacorona virus
PEDV: Porcine epidemic diarrhea virus
PPV: Porcine parvovirus
PRV: Pseudorabies virus
RV: Rotavirus
TTSuV1: Torque teno sus viruse type 1
TTSuV2: Torque teno sus viruse type 2
